# Environment-Friendly Preparation and Characterization of Multilayered Conductive PVP/Col/CS Composite Doped with Nanoparticles as Potential Nerve Guide Conduits

**DOI:** 10.3390/polym16070875

**Published:** 2024-03-22

**Authors:** Aleksandra Sierakowska-Byczek, Julia Radwan-Pragłowska, Łukasz Janus, Tomasz Galek, Karol Łysiak, Mirosław Tupaj, Dariusz Bogdał

**Affiliations:** 1 Department of Biotechnology and Physical Chemistry, Faculty of Chemical Engineering and Technology, Cracow University of Technology, Warszawska 24 Street, 31-155 Cracow, Poland; lukasz.janus@pk.edu.pl (Ł.J.); pcbogdal@pk.edu.pl (D.B.); 2 Faculty of Mechanics and Technology, Rzeszow University of Technology, Kwiatkowskiego 4 Street, 37-450 Stalowa Wola, Poland; t.galek@prz.edu.pl (T.G.); k.lysiak@prz.edu.pl (K.Ł.); mirek@prz.edu.pl (M.T.)

**Keywords:** multifunctional polymers, responsive polymers, bio-based polymers, nanocomposites, nerve guidance conduits

## Abstract

Tissue engineering constitutes the most promising method of severe peripheral nerve injuries treatment and is considered as an alternative to autografts. To provide appropriate conditions during recovery special biomaterials called nerve guide conduits are required. An ideal candidate for this purpose should not only be biocompatible and protect newly forming tissue but also promote the recovery process. In this article a novel, multilayered biomaterial based on polyvinylpyrrolidone, collagen and chitosan of gradient structure modified with conductive nanoparticles is presented. Products were obtained by the combination of electrospinning and electrospraying techniques. Nerve guide conduits were subjected to FT-IR analysis, morphology and elemental composition study using SEM/EDS as well as biodegradation. Furthermore, their effect on 1321N1 human cell line was investigated by long-term cell culture. Lack of cytotoxicity was confirmed by XTT assay and morphology study. Obtained results confirmed a high potential of newly developed biomaterials in the field of nerve tissue regeneration with a special focus on injured nerves recovery.

## 1. Introduction

Peripheral nerve injuries can be divided according to the degree of damage. Mild injuries are characterized by lack of disruption of nerve continuity, which manifests itself in deteriorated nerve conduction, sensory disturbances, and muscle weakness, but the symptoms disappear spontaneously in a relatively short time. In the case of serious injuries, the continuity of the nerve is interrupted, which requires surgical intervention [1].

Autologous transplantation is considered to be the gold standard, but its disadvantages include, among others, limited availability of tissue or the creation of another wound at the donor site. Another solution is allogeneic transplantation, but it involves the use of immunosuppressive drugs and other limitations. The solution to eliminate the defects of transplants is the use of synthetic nerve channels. Currently used nerve channels can be divided into non-resorbable and resorbable ones. Non-resorbable channels made of polytetrafluoroethylene provide mechanical support for the regenerating tissue, but another surgery is necessary to remove the material. Resorbable channels include these produced, among others, from collagen or polyglycolic acid, however, they have many drawbacks such as poor mechanical strength and uncontrolled biodegradation time. The limitations of both non-resorbable and resorbable nerve channels include also low effectiveness and lack of additional stimulation of tissue regeneration [2,3].

Biomaterials used for tissue reconstruction and regeneration should provide appropriate environmental conditions for cells, support their viability and specific functions. This can be achieved by using polymer membranes that are able to act as an extracellular matrix when they have specific morphology and physicochemical properties. Membrane biomaterials ensure the transfer of nutrients and cellular metabolites through their porous structure. The nanofibrous layers are characterized by a larger specific surface, porous structure, permeability, and better mechanical properties. They can be produced from polymers that are easily electrospun, e.g., polyethylene oxide (PEO), polylactic acid (PLA), polyvinyl alcohol (PVA), or polyvinylpyrrolidone (PVP) [1,2,3,4].

PVP is one of the most frequently used vinyl polymers in medicine due to its good stability, bio- and hemocompatibility, lack of antigenicity, biodegradability, low cytotoxicity, and the ability to complex both hydrophilic and hydrophobic substances. Additionally, low molecular weight PVP easily penetrates biological membranes, so it does not adhere to body tissues. Thanks to these properties, it has been approved by the US Food and Drug Administration as a safe polymer for biological experiments. Due to its physicochemical properties, such as high chemical and thermal resistance, solubility in water and other non-toxic organic solvents, it can be easily processed into the form of nanofibers using electrospinning technique [5,6,7,8,9,10,11,12,13]. 

In order to improve the biological properties of nanofibrous membranes, it is possible to add additional layers using electrospraying from biopolymers, e.g., chitosan and collagen, for which processing is difficult due to their chemical structure, lack of solubility or degradation in solvents commonly used in electrospinning [14,15,16,17].

Collagen is a protein that is the main component of the extracellular matrix. It is present in most tissues of all animal organisms, including bones, skin, and tendons. Its primary task is to maintain the structure, and to ensure elasticity and tensile strength. Due to low toxicity, or antigenicity, high biocompatibility, bioactivity and biodegradability of the materials, it is widely used in different fields of regenerative medicine. However, due to its thermolability, there are serious problems with its processing and with obtaining more complex structures, which strongly limits its commercial use in more sophisticated applications [18,19,20,21].

Chitosan is a derivative of chitin, which is obtained from sources such as: the external shells of marine crustaceans, external skeletons of insects or cell walls of fungi, and is considered, after cellulose, to be the most abundant natural polymer. Chitosan is non-toxic, biocompatible, biodegradable, and can be relatively easily modified both chemically and enzymatically. It has also been shown that chitosan has antibacterial properties by acting on the cytoplasmic membrane of bacterial cells. These properties make chitosan useful in medicine and various industries [22,23,24,25,26].

In the electrospinning and electrospraying processes, the polymer solution is charged electrically using a high-voltage power supply, which leads to the liquid drop formation at the end of the spinneret which changes into a cone, called a Taylor cone. Then the solvent evaporates, and the electrically charged fibers or microspheres are attracted towards the oppositely charged collector. The electrospinning and electrospraying processes are affected by parameters such as the average molar mass of the polymer, electric voltage, the properties of the solution (viscosity, conductivity, and surface tension), and liquid flow rate [27].

Electrospinning and electrospraying techniques allow biomaterials to be obtained with high efficiency in a short time, using non-toxic solvents, which can also be easily recovered. Additionally, it is possible to control the size of newly-formed materials, which reduces the waste generated during the process. Thanks to these features, these techniques are environmentally friendly and consistent with the principles of green chemistry [28,29].

The use of the right type of nanoparticles in tissue engineering can significantly improve the biological, mechanical, and electrical properties of the scaffolds. In biomedical applications, it is particularly important to prevent microbiological contamination of the biomaterials used for implantation due to the risk of infection. This can be achieved by using certain metal nanoparticles with antimicrobial activity. For example, it has been shown that silver nanoparticles are able to fight some antibiotic-resistant bacteria. Another advantage of using nanoparticles in biomaterials is the fact that they increase the rate of cell proliferation and affect the differentiation of mesenchymal stem cells [28,29,30,31,32].

Additionally, nanocomposites with metal nanoparticles show better mechanical properties compared to scaffolds alone. This is due to the formation of new bonds (mainly hydrogen) between the nanoparticles and the polymer matrix. In addition, conductive nanoparticles enable the stimulation of nerve cells using an electric field, which makes it possible to restore normal tissue functions [32,33,34,35].

There are many methods of nanoparticle synthesis, which are characterized by limitations such as lack of repeatability of shapes and sizes, lack of stability, the need to use high temperatures, long reaction times, and low efficiency. A method that does not have these drawbacks is synthesis in a microwave radiation field, which is consistent with the principles of green chemistry since it reduces the energy consumed.

The main aim of this work was to obtain a hybrid material which would combine the conductive nature of PVP and metallic nanoparticles together with superior bioactivity and biocompatibility of natural polymers such as collagen and chitosan. The following approach resulted in the preparation of composite NGCs which were subjected to physicochemical and biological properties evaluation to verify their potential in nerve injury therapy via tissue engineering by meeting the requirements for nerve guide conduits such as lack of cytotoxicity, and regenerated nerve protection, as well as a capacity for electrostimulation and electric impulse transfer.

## 2. Materials and Methods

### 2.1. Materials

Polyvinylopyrrolidone (PVP; Mw 40000), collagen type I, chitosan, acetic acid 99%, ethanol, silver nitrate, chloroauric acid, chloroplatinic acid, sodium citrate, Dulbecco’s Modified Eagle Medium (DMEM) with glucose content cell culture medium, fetal bovine serum (FBS), phosphate buffer solution (PBS), CaCl_2_·2H_2_O, MgSO_4_, KCl, KOH, KH_2_PO_4_, Na_2_HPO_4_, NaHCO_3_, NaCl, HCl, NaOH, mouse fibroblasts L929 cell line, 1321N1 Cell Line human glial cells for commercial use (The European Collection of Authenticated Cell Cultures (ECACC)), trypsin with EDTA, antibiotics (streptomycin/penicillin), cell proliferation kit XTT were purchased from Sigma Aldrich, Poznań, Poland.

### 2.2. Methods

#### 2.2.1. Preparation of Conductive Nanoparticles

Gold, silver, and platinum nanoparticles were obtained by reducing silver nitrate, chloroauric acid and chloroplatinic acid. The concentrations of the solutions were AgNO₃ 200 mg/250 cm^3^ H_2_O, HAuCl_4_∙3H_2_O 215 mg/250 cm^3^ H_2_O, H_2_(PtCl_6_)∙6H_2_O 300 mg/250 cm^3^ H_2_O. To reduce the nanoparticles sodium citrate was used. Syntheses were carried out according to variable parameters given in Table 1 using an Ertec MAGNUM II microwave reactor (ERTEC-POLAND Edward Reszke, Wrocław, Poland). Next, the nanoparticles were purified from unreacted substrates using dialysis tubes for 48 h. Then, using Atomic Absorption Spectroscopy (Philips PU-9100X), the efficiency of the nanoparticle synthesis reaction was determined to be 100% and the solutions were diluted to a concentration of 0.2 mg/mL.

#### 2.2.2. Preparation of NPs/PVP/ Col/CS Nanocomposites

**Figure 1:**
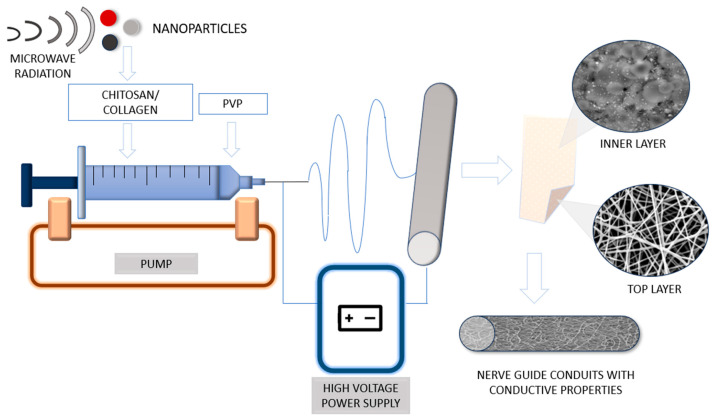
General scheme of obtaining nerve guidance conduits.

Firstly, a 10% PVP aqueous solution was prepared. To dissolve biopolymers, acetic acid solution was prepared from concentrated acetic acid and aqueous solutions of Ag, Au or Pt nanoparticles suspensions. Chitosan and collagen were dissolved at a concentration of 2% in a 5% acetic acid solution, containing an aqueous solution of one of three types of metallic NPs. Ethanol was added to the PVP, chitosan and collagen solutions in a 1:1 volume ratio. The materials were obtained using a self-made device, using a high-voltage power supply from LEYBOLD and a syringe pump from NEW ERA PUMP SYSTEMS model Infusion ONE NE-300, the voltage was 25.5 kV, the distance between the needle and the collector was 5 cm, the flow rate of the solution was 0.15 mL/min. General pathway of their obtainment is given in Figure 1.

#### 2.2.3. Chemical Structure Study

Chemical structure was evaluated using Fourier-transform infrared spectroscopy (FT-IR) equipped with ATR adapter. For the experiments, Thermo Nicolet Nexus 470 FT-IR spectrometer (Thermo Fisher Scientific, Waltham, MA, USA) was used. XRD analysis has been carried out using X-ray Diffractometer (Empyrean, PANalytical, Almelo, Netherlands).

#### 2.2.4. Conductivity Study

The conductivity of purified nanoparticle aqueous solutions was measured using the ELMETRON CC-411 conductivity meter. The conductivity of the ready membranes was determined using ELMETRON CX-742 equipped with graphite electrodes with a surface area of 1 cm^2^. Materials with a thickness of 0.1 mm were previously swollen in PBS solution. Tests were carried out at a room temperature. 

#### 2.2.5. TEM Analysis

Nanomaterials were investigated by Transmission Electron Microscope purchased from Jeol, (Peabody, MA, USA). PDI index was calculated with Excel software (Office 365 Education 2019) using data generated from TEM images using Fiji J software ImageJ2.

#### 2.2.6. SEM Analysis

Ready products were investigated by FEI Quanta 650 FEG Scanning Electron Microscope purchased from FEI (ThermoFisher Scientific, Eugene, OR, USA). Prior to analysis, samples were sputtered with copper. 

#### 2.2.7. Cytotoxicity Study

The human astrocyte cell line 132N1 and the L929 mouse fibroblasts cell line, which is commonly used in cytotoxicity tests of biomaterials in accordance with PN-EN ISO 10993-5:2009 standard (Biological evaluation of medical devices Part 5: Tests for in vitro cytotoxicity) [36], were used for the experiments. The cell culture was conducted for 168 h (132N1) and 48h (L929) under standard conditions (5% CO_2_ concentration, high humidity, 37 °C). DMEM medium (Gibco, Waltham, MA, USA), enriched with fetal bovine serum at a concentration of 10% and an antibiotic, was changed every 48 h. The observation of cells morphology under inverted microscope was performed using 40× magnification (Delta Optical IB-100 microscope, Planeta Oczu, Zielona Góra, Poland). The XTT assay was used to determine cell viability by measuring absorbances at 450 nm according to the producer’s protocol (Roche, Basel, Switzerland). 

#### 2.2.8. Biodegradation

Biodegradation studies were carried out using human lysozyme and collagenase. Each time, the dried and weighed sample was placed in solutions of SBF, human lysozyme at a concentration of 10 mg/L, and collagenase at a concentration of 2 g/L, respectively. These concentrations correspond to the concentrations in the human body. After specified intervals, the samples were removed from the solution, dried, and weighed. The progress of biodegradation was determined by calculating the weight loss of each sample according to the following equation:

BD = (W_0_ − W_t_)/W_0_ × 100%

where

BD—biodegradation degree,
W_0_—the initial weight,
W_t_—the weigh after time t.

#### 2.2.9. Antibacterial Properties

Minimal inhibitory concentration (mic) was tested. From 2 to 100 μL of solutions of Au, Pt, Ag nanoparticles were added to the bacterial suspension. Absorbances were measured at 630 nm after 2, 6, 12 and 24 h of incubation at 37 °C. The research was conducted on selected strains of bacteria: *S. aureus* and *P. aeruginosa*.

## 3. Results

**Figure 2:**
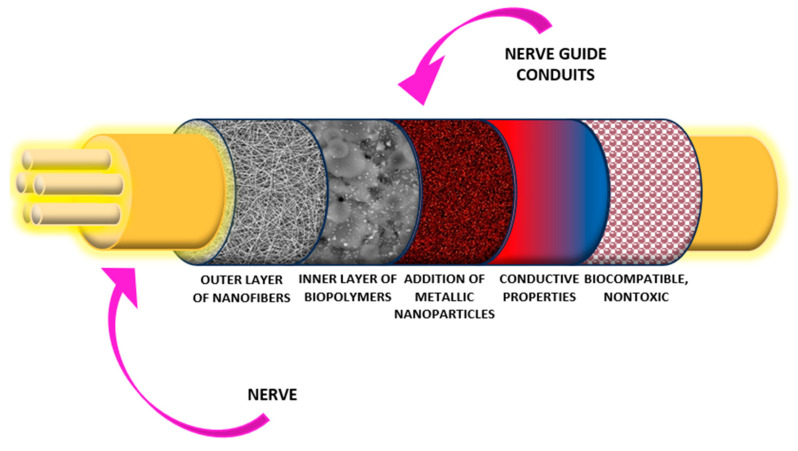
General scheme of newly prepared NGCs.

As a result of a combination of electrospinning and electrospray, a series of hybrid biomaterials were prepared according to the scheme given in Figure 2, combining a nanofibrous PVP external layer and a composite collagen/chitosan layer containing three different types of NPs (Ag, Au or Pt), to provide satisfactory conductivity together with superior biocompatibility and bioactivity, as well as the potential for electro-stimulation.

### 3.1. Nanoparticles Characteristics

**Figure 3:**
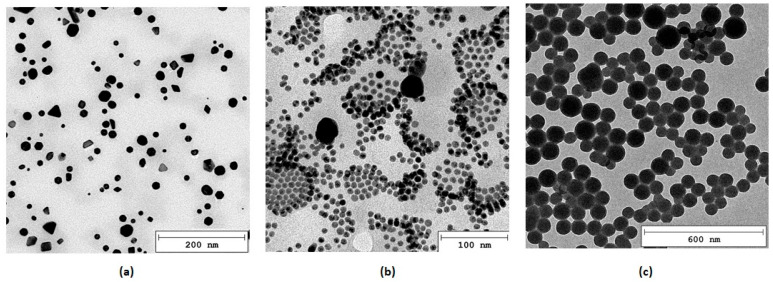
TEM microphotographs of the nanoparticles: (**a**) Au; (**b**) Ag; (**c**) Pt.

Modern biomaterials should not only meet basic requirements for medical devices such as a lack of cytotoxicity but also exhibit additional features which would accelerate recovery process. New tissue formation acceleration can be achieved by various methods. Among them electrostimulation can be distinguished. For this reason, three types of metallic nanoparticles were obtained, namely gold, silver and platinum, which are characterized by excellent conductivity. As shown in Figure 3, all prepared NPs were characterized by nanometric size below 100 nm and spherical morphology. Particle size distribution analysis revealed that PDI for Au NPs was 0.104, for Ag NPs 0.018 and for Pt NPs 0.041, respectively. Importantly, their diameter exceeded 10 nm which makes them a good candidate for biomaterials components since they are unlikely to bioaccumulate in organs such as the liver or kidneys [34]. 

### 3.2. FT-IR Chemical Structure Analysis

**Figure 4:**
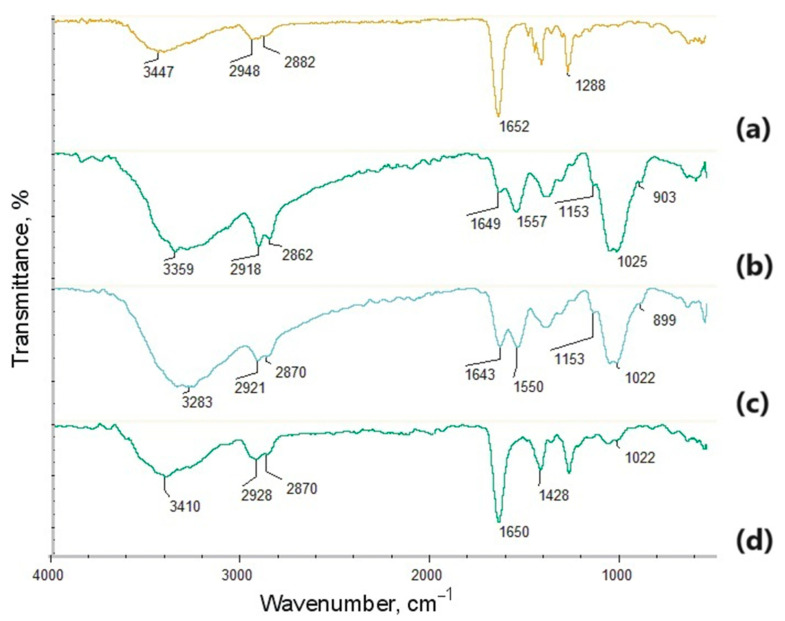
FT-IR spectrum of the (**a**) PVP; (**b**) chitosan; (**c**) chitosan/collagen; (**d**) PVP/chitosan/collagen.

The aim of this research was to prepare advanced biomaterials capable of cells stimulation and nerve regeneration. For this reason, multilayered tubes were obtained using a combination of electrospinning and electrospraying techniques to produce conduits of gradient structure and advanced propertied in six different arrangements. Aforementioned methods enable processing of even thermolabile substrates such as collagen which is known for its superior biocompatibility. As shown in Figure 2, potential NGCs were built from different layers. The internal layer was prepared using an electrospraying process from the PVP/chitosan/collagen composite, modified with newly prepared nanoparticles (Ag, Au or Pt), to combine the excellent cytocompatibility and the bioactivity of the chitosan and the natural extra cellular matrix component together with PVP. The presence of NPs enabled the maintenance of conductive properties of the material, and the possibility of electric pulse delivery directly to injured nerves under recovery to stimulate the process. The external layer was prepared from PVP nanofibers, for which the FT-IR spectrum is presented in Figure 4a. It can be noticed that polymer processing via electrospinning did not cause chemical degradation and no impurities or solvent leftovers can be distinguished in the layer, which can be confirmed by the presence of a broad band corresponding to N-H groups present in amide rings at 3447 cm^−1^, bands at 2948 cm^−1^ and 2882 cm^−1^ typical of -CH_3_ and -CH_2_- groups, a sharp band at 1652 cm^−1^ typical of C=O stretching vibrations, and C-N moieties at 1288 cm^−1^ [34]. The spectrum visible in Figure 4b corresponds to the layer prepared via electrospray from chitosan. Again, no degradation is visible although reactive, highly concentrated acetic acid was used, which can be confirmed by bands corresponding to 1,4-β-glycosidic bonds at 1025 cm^−1^ and the glucopyranose ring at 903 cm^−1^. Moreover, typical bands corresponding to free hydroxyl groups at 3359 cm^−1^ can be observed, as well as at 2918 cm^−1^ and 2862 cm^−1^ coming from aliphatic moieties (-CH_3_, -CH_2_-), and 1649 cm^−1^ typical for amide bonds in *N*-acetylglucosamine units. Finally, bands characteristic to free amino groups are visible at 1557 cm^−1^ and 1153 cm^−1^, which are responsible for the favorable chitosan biological properties such as cell adhesion or interaction with biomolecules affecting proliferation, and as a consequence tissue regeneration [35]. Figure 4c reveals the FT-IR spectrum of the composite layer containing two biopolymers, namely chitosan and collagen. Again, signatures for chitosan bands are visible at 3283 cm^−1^ coming from hydroxyl groups, 2921 cm^−1^, 2870 cm^−1^ typical for -CH_3_ and -CH_2_- groups, and 1550 cm^−1^/1153 cm^−1^ corresponding to glycosidic bonds, and glucopyranose rings (1022 cm^−1^ and 899 cm^−1^). The presence of the collagen can be confirmed by the increased intensity of the band coming from peptide bonds at 1643 cm^−1^ [37]. The last spectrum presents the chemical structure of the layer prepared from all three polymers. The presence of PVP can be confirmed by the intense band at 3410 cm^−1^, typical of amides present in a pyrrolidone mer. Bands corresponding to chitosan and collagen are present at 1650 cm^−1^ (overlapping amide with carbonyl group), and 1022 cm^−1^ (glycosidic bonds in chitosan between the mers).

**Figure 5:**
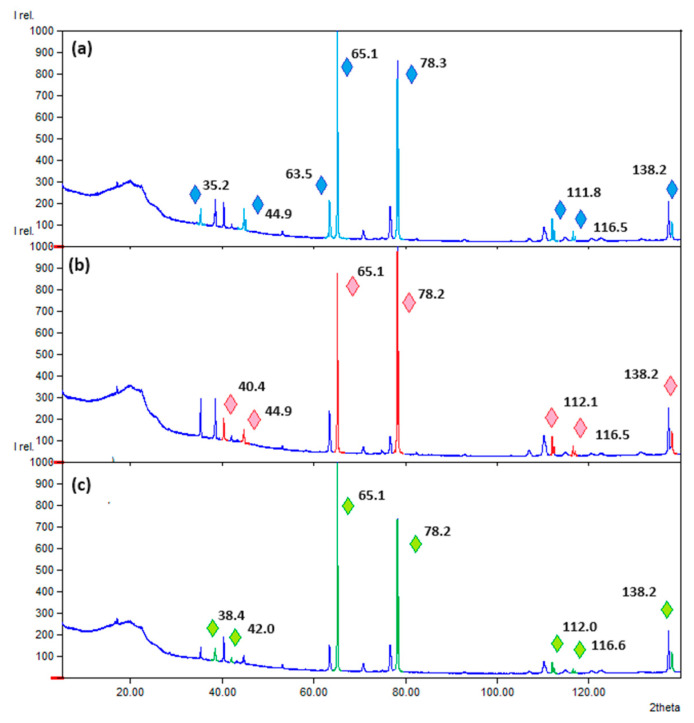
XRD analysis of the (**a**) PVP/Col/CS Ag NPs; (**b**) PVP/Col/CS Au NPs; (**c**) PVP/Col/CS Pt NPs. Reflexes corresponding to metal NPs are shown in different colors.

To investigate composite layer PVP/chitosan/collagen containing nanoparticles XRD analysis was performed (Figure 5). As can be noticed, all three diffractograms reveal reflexes typical for metallic nanoparticles (Ag, Au and Pt, respectively), which proves their incorporation inside the polymeric matrix. Due to the nature of spherical nanoparticles, no signal crystallites can be distinguished [38].

### 3.3. Conductivity Study

**Figure 6:**
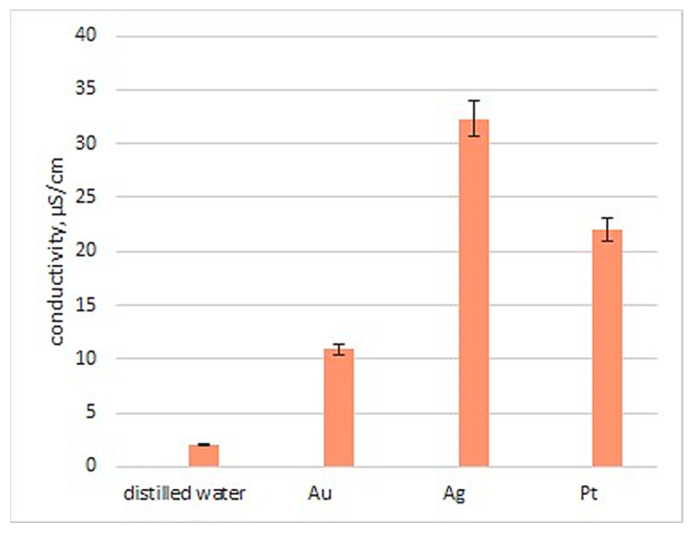
Conductivity of the prepared nanoparticles.

**Figure 7:**
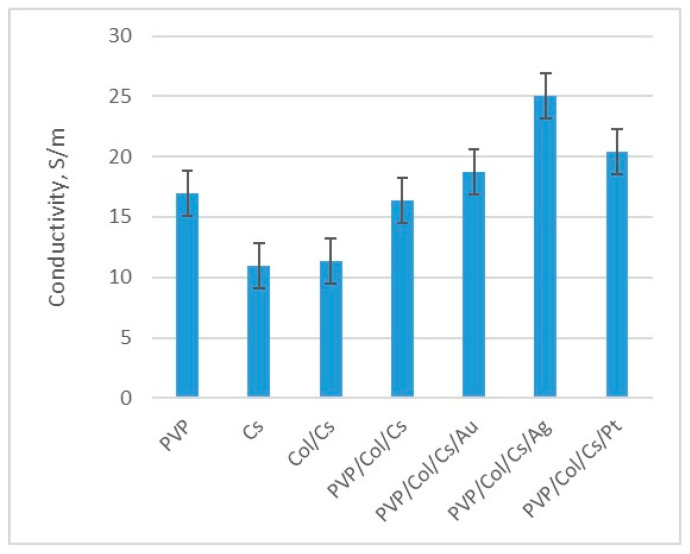
Conductivity of the prepared materials.

Conductivity is a highly desired feature for biomaterials dedicated to nerve tissue engineering applications since it provides the possibility for electrostimulation and regeneration process enhancement. Gold, silver and platinum nanoparticles are known for their ability to conduct electricity due to their metallic nature. However, this property may depend on the synthesis pathway as well as NP size and morphology. As shown in Figure 6, all of the prepared nanomaterials exhibited conductivity. The study revealed that the highest value of this parameter was obtained for the solution of silver nanoparticles (32 µS/cm), followed by platinum and finally gold (11 µS/cm). Synthesized NPs were further used during multi-layered NGC preparation. As shown in Table 2, six different nerve guidance channels were obtained which varied by chemical composition. First NGCs were prepared via an electrospinning process using PVP as a raw material. PVP is one of the most widely used conductive polymers due to its low cytotoxicity and processability. Figure 7 reveals that the nanofibrous channel based on pure polyvinylpyrrolidone exhibited satisfactory conductivity, compared to the natural polymers, namely chitosan and collagen. Nevertheless, these macromolecular compounds are known to have superior biocompability and bioactivity, therefore it is necessary to combine all these features. Thus, to enhance chitosan and collagen’s original ability for electron transmission, before electrospray processing, their solutions were doped with three types of metallic NP solutions. As shown in Figure 7, this approach resulted in the obtainment of the nanocomposities with more satisfactory properties and higher conductivity, compared to the three polymeric component composites alone (PVP/Col/CS). As can be noticed, all nanocomposites containing three different NPs exhibited higher electrical properties. The addition of nanoparticles significantly improved the conductive properties of the materials, with an increase of 14% for gold nanoparticles, 52% for silver and 24% for platinum. Their use increased the conductive properties of the materials by 44% compared to the Col/Cs composite. Such conductivity meets the requirements of biomaterials capable of regenerating tissue by electrostimulation, since it should exceed 8 µS/cm.

**Table 2:** Samples description and composition.

Sample	Polymers	Nanoparticles
1.	PVP	-
2.	PVP/CS	-
3.	PVP/Col/CS	-
4.	PVP/Col/CS	Au
5.	PVP/Col/CS	Ag
6.	PVP/Col/CS	Pt

### 3.4. SEM

**Figure 8:**
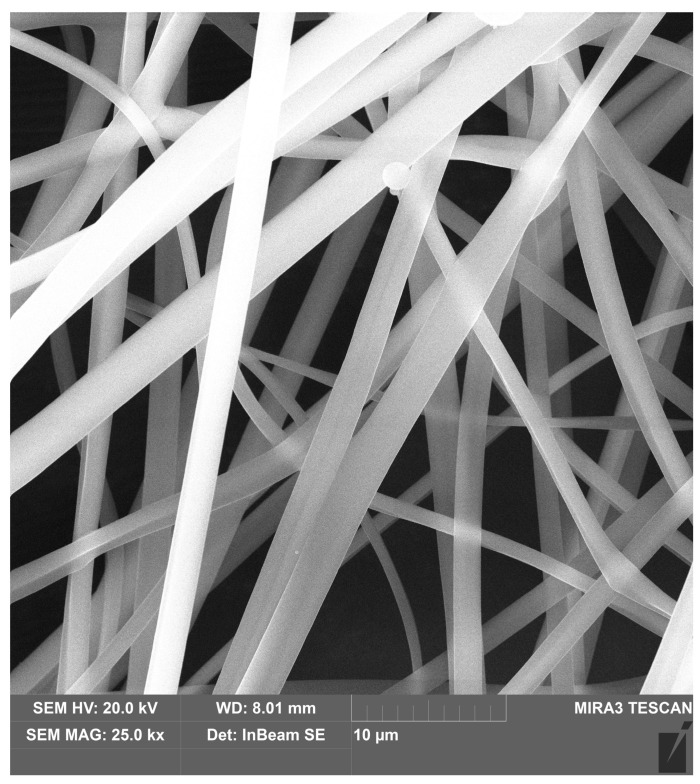
SEM microphotographs of the PVP nanofibers, magnification 500,000×.

The SEM analysis showed that the obtained PVP fibers are homogeneous, with a nanometer thickness, without defects and thickenings (Figure 8). The structure of the layer is characterized by high porosity, thanks to the irregular arrangement of the fibers. Such morphology promotes cells adhesion and proliferation. It also provides nutrients and biomolecules such as growth factors delivery and gas exchange (O_2_/CO_2_). PVP nanofibers constitute external layer of the NGCs since they will provide optimal parameters for nerve regeneration and facile delivery of necessary compounds. Due to its satisfactory conductivity this layer can act as a platform for biomolecules transport such as growth factors as well as cells migration to recovery site and receive electrical impulses and pass them to internal layer.

**Figure 9:**
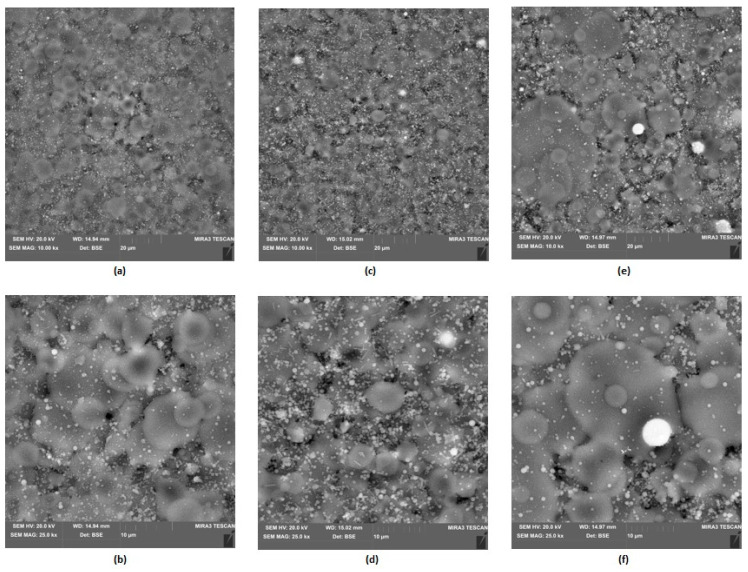
SEM microphotographs of the Col/Cs layers doped with NPs: (**a**) Au ; (**b**) Au; (**c**) Ag; (**d**) Ag; (**e**) Pt; (**f**) Pt.

**Figure 10:**
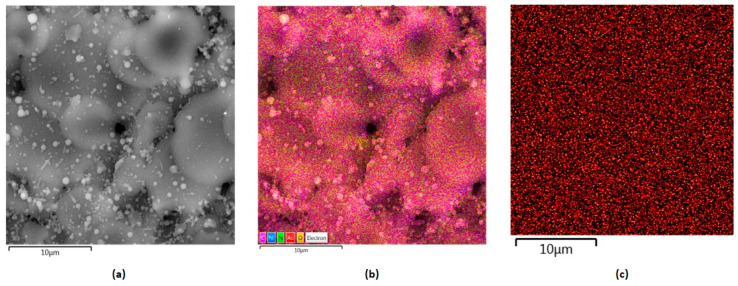
SEM microphotographs of the nanocomposites with elemental mapping: (**a**) Col/Cs/Au, magnification 500,000×; (**b**) elemental mapping; (**c**) Au mapping.

**Figure 11:**
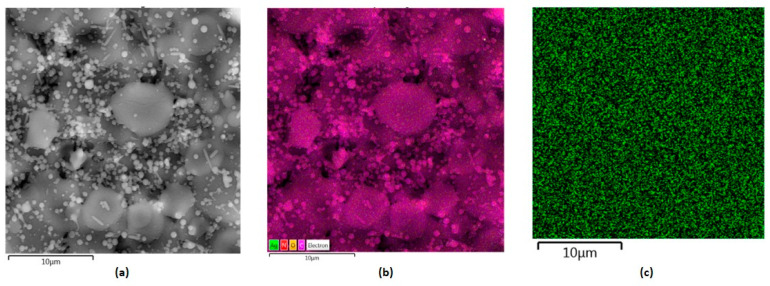
SEM microphotographs of the nanocomposites with elemental mapping: (**a**) Col/Cs/Ag, magnification 500,000×; (**b**) elemental mapping; (**c**) Ag mapping.

**Figure 12:**
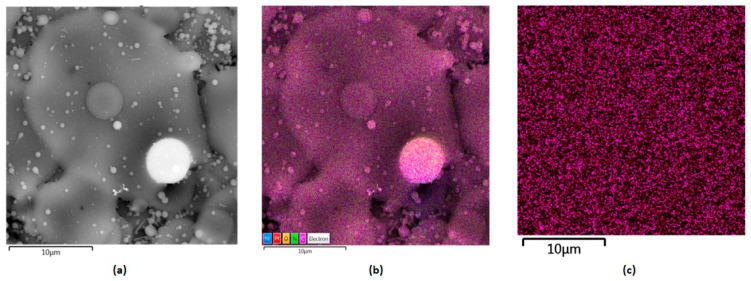
SEM microphotographs of the nanocomposites with elemental mapping: (**a**) Col/Cs/Pt, magnification 500,000×; (**b**) elemental mapping; (**c**) Pt mapping.

Figure 9 shows layers prepared by the electrospray technique containing collagen, chitosan and metallic nanoparticles. They constitute the inner part of the NGC, which plays a protecting role from external factors as well as offering mechanical support for peripheral nerve regeneration. Their slight porous/grooved morphology enables structural integrity, which provides the possibility to guide regenerating tissue and to stimulate the process. This is due to their chemical composition, namely the presence of chitosan and collagen, known for their bioactivity and their positive impact on cell proliferation and maturation. Electrical stimulation is found to be an interesting yet effective way of peripheral nerve injury management. Therefore, the internal layer composed of biopolymers was further modified with highly conductive nanoparticles, namely gold, silver and platinum. Figure 10, Figure 11 and Figure 12 show SEM images of Col/Cs/nanocomposites with elemental mapping. This analysis confirmed the uniform modification of the materials with aforementioned NPs, which provides good conductivity in the layer and the possibility of homogenous stimulation via electric impulses to improve biochemical cues during nerve recovery.

**Figure 13:**
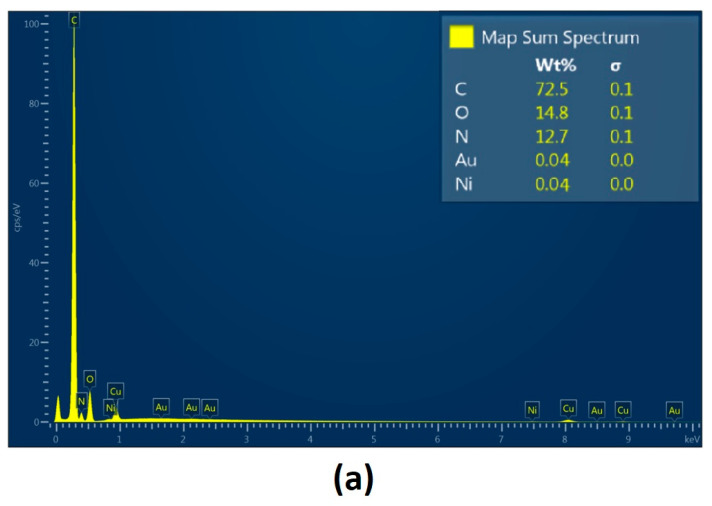

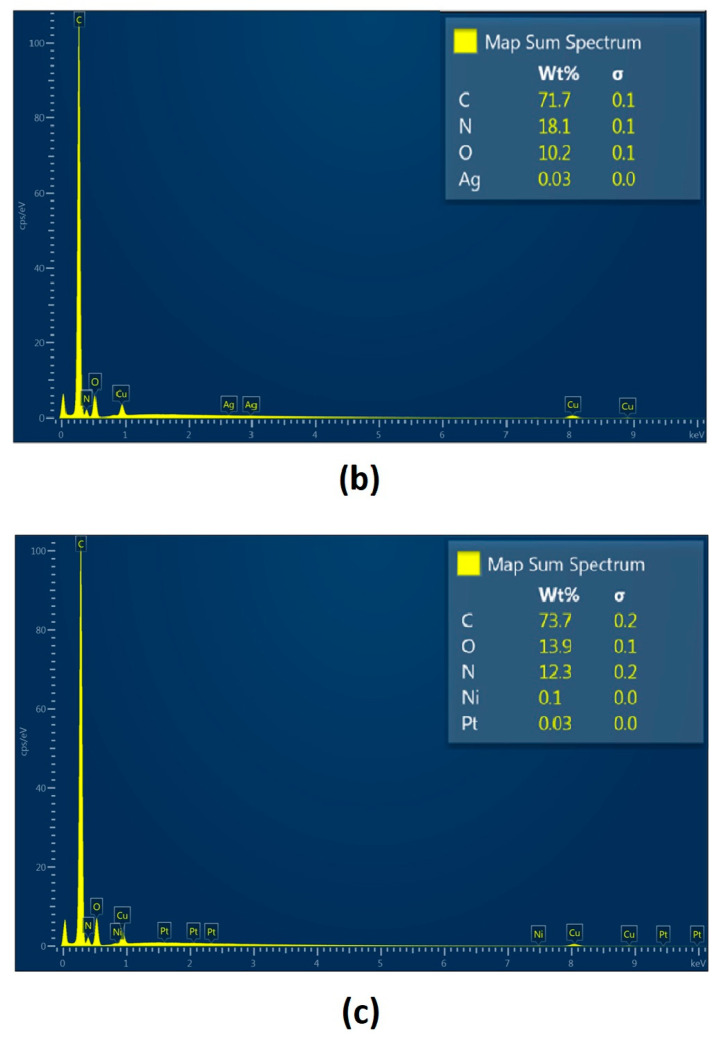
EDS analysis of the: (**a**) Col/Cs/Au; (**b**) Col/Cs/Ag; (**c**) Col/Cs/Pt. Cu presence is a consequence of sample sputtering for SEM imaging.

As shown in Figure 13, according to EDS analysis, the weight percentage content of the metallic NPs was 0.04% for Au nanoparticles and 0.03% for silver and platinum, respectively.

### 3.5. Biodegradation 

**Figure 14:**
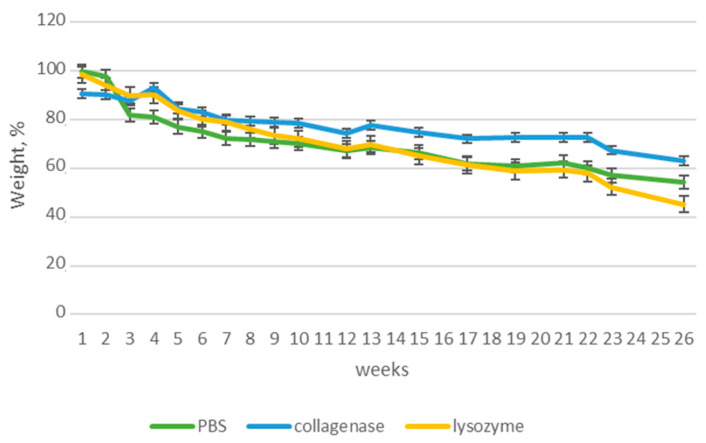
Biodegradation of PVP/Col/Cs.

**Figure 15:**
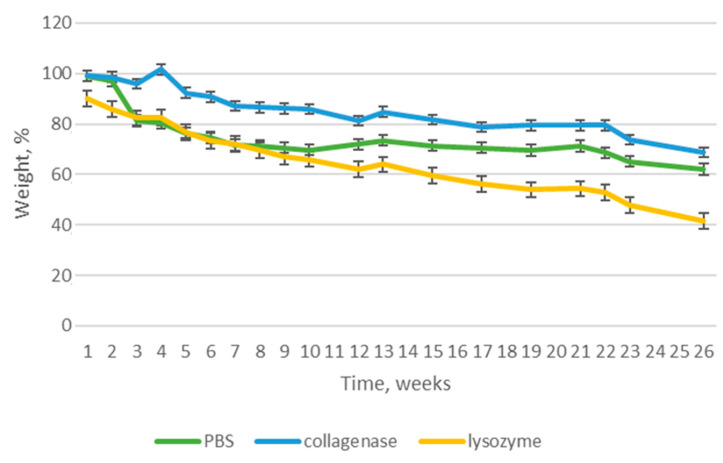
Biodegradation of PVP/Col/Cs/nano Au.

**Figure 16:**
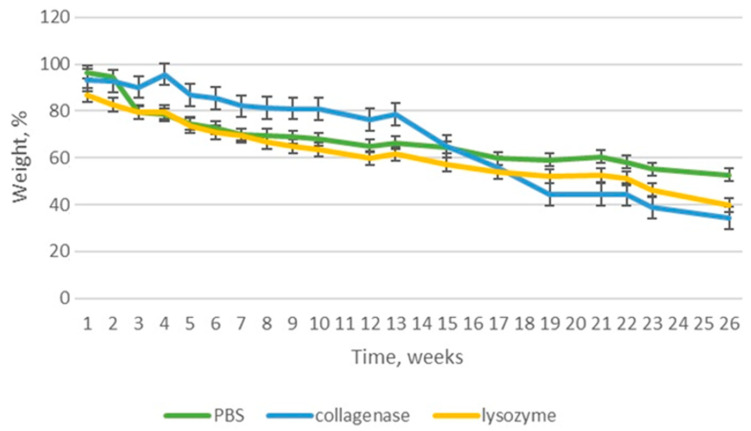
Biodegradation of PVP/Col/Cs/nano Ag.

**Figure 17:**
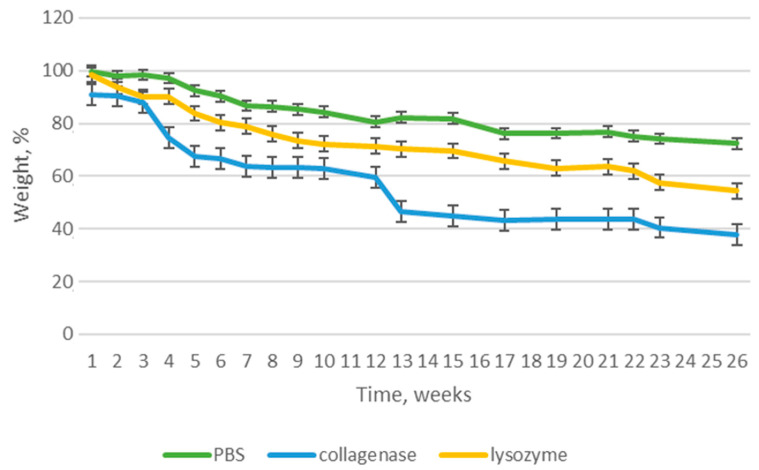
Biodegradation of PVP/Col/Cs/nano Pt.

Newly-developed nerve guide conduits are dedicated to peripheral nerve injuries management and constitute an alternative to traditional treatment methods based on transplantation. They should not only protect from external biological, chemical and mechanical factors, but also act as a template for newly formed tissue, support the regeneration process and stimulate it. Although the NGC-based treatment strategy is a long-term approach, the biomaterial should undergo biodegradation in time and decay while the nerve is rebuilt. To verify stability of the novel NGCs under in vivo simulated conditions, susceptibility to aqueous environment as well as the presence of lysing enzymes was verified over a 26-week period. Figure 14, Figure 15, Figure 16 and Figure 17 reveal results of this biodegradation study. The products were maintained in human body-like conditions. Their weight loss with time was investigated in pure PBS as well as PBS containing collagenase and lysozyme. It should be noticed that biomaterial stability varied depending on the chemical composition and type of NPs. In all cases, the weight loss was linear and mostly stable in time. 

The sample containing the inner layer composed from polymers alone (Figure 14) biodegraded almost 60% in the solution containing lysozyme, which shows that glycosidic bonds are most prone to enzymatic hydrolysis. Interestingly, weight decrease (around 40%) was noticed for collagenase, which is responsible for peptide bond breakage. Degradation in PBS was not affected by any enzyme, leading to almost 50% weight decrease. 

Figure 15 presents the results of biodegradation after 6.5 months for NGCs with PVP/Col/Cs/nano Au layers. The weight loss was similar to PVP/Col/Cs alone as well as correlation between medium composition and susceptibility to degradation. Nevertheless, weight loss was slightly higher than the previous sample, which suggests that Au NPs could somehow accelerate enzymatic hydrolysis of chitosan due to catalytic properties of the nanoparticles, agreeing with the data of other researchers, suggesting that gold NPs affect such reactions [39].

Figure 16 revels the biodegradation progress obtained for NGCs with PVP/Col/Cs/nano Ag layers. Again, the weight loss was like the PVP/Col/Cs sample; however, it has been noticed that enzymatic activity was higher than previously, and the Ag NPs presence stimulates both lysozyme and collagenase-driven amide and glycosidic bond breakage. Also, it can be noticed, that collagenase-induced biodegradation significantly accelerated after the 13th week, which can be explained by higher availability of peptide moieties probably due to a lower number of hydrogen bonds or other inter- and intra-molecular interactions.

Figure 17 shows the results of the biodegradation study for the NGC containing Pt nanoparticles in its inner layer. In this case, similarly to the PVP/Col/Cs/nano Ag sample, the highest weight loss was measured for the sample immersed in a medium containing collagenase and exceeded 60%. Interestingly, significant differences between degradation solutions can be noticed, that is around 20% (40% between PBS and collagenase). Also, as for the Ag NP-containing sample, sudden weight loss arose between 12 and 13 weeks. It is noteworthy that the degradation process in the presence of collagenase was quite irregular and unstable. Additionally, this biomaterial was characterized by the highest stability over time in pure PBS (weight loss above 20%).

### 3.6. Determination of the Cytotoxicity of the Obtained Nanocomposites

An ideal biomaterial should meet numerous requirements among which the most important is biocompatibility. To provide this feature, for novel NGC preparation a dual approach was undertaken. For the fabrication of the outer nanofibrous layer conductive PVP was used, whereas to obtain the inner one a combination of chitosan and collagen was used, further doped with metallic NPs to enable electrostimulation with direct or alternating current. Moreover, biomaterials have a dual nature due to their two different layers with varying characteristics, purpose, and architecture.

**Figure 18:**
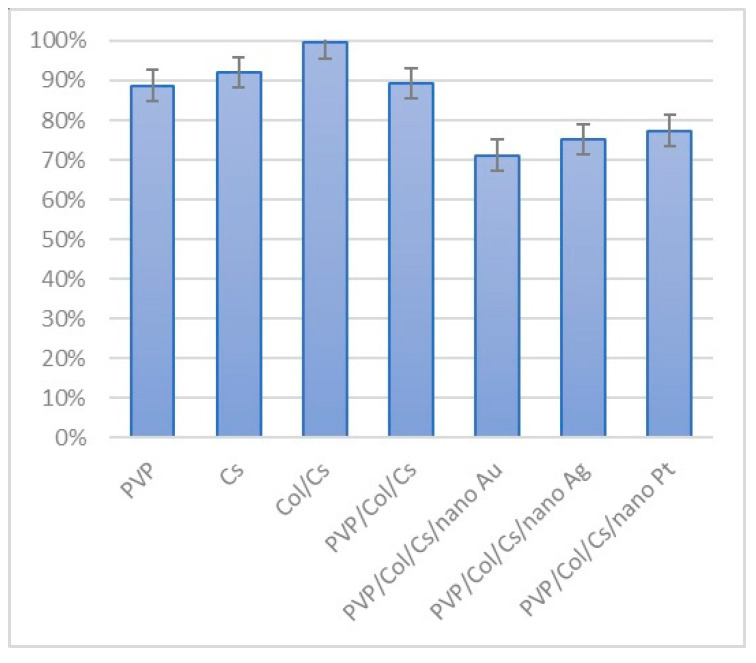
XTT assay results carried out on L929 mouse fibroblasts.

To investigate their biocompability, two different tests were carried out using 132N1 cells, using human astrocytes and L929 mouse fibroblasts, which are recommended by PN-EN ISO 10993-5:2009 standard (Biological evaluation of medical devices Part 5: Tests for in vitro cytotoxicity), Polski Komitet Normalizacyjny, Warsaw, Poland 2009 [36] for biological properties of medical devices assessment. Figure 18 presents results of XTT assay which provides quantitative data regarding cell viability and proliferation since it is based on the enzymatic activity during glycolysis and NAD(P)H production. According to the ISO-10993 standard, metabolically active cells should constitute at least 70% compared to cells cultured without evaluated biomaterial. As shown in the Figure 18, all the tested samples can be described as not cytotoxic, yet their impact on fibroblasts is diverse. Firstly, for PVP nanofibers, cell viability was slightly lower than the control, thus the assumption that the outer layer composed of this material can both protect newly forming tissue and act as a template was correct. To verify precisely which combination of the inner layer was directly involved in peripheral nerve regeneration, tubes were prepared for testing by electrospray from pure chitosan, a mix of chitosan and collagen, as well as a mix of all three polymers. It can be noticed that pure chitosan and the mix of PVP, CS and Col layers exhibited cell viability similar to PVP alone, whereas the combination of CS with collagen resulted in an amount of metabolically active cells at the same level as the control. Nevertheless, the difference is negligible. No increased proliferation activity was observed during the standard cell culture. Further study involved determination of the fibroblast response to the NP-doped inner composite layer, whose presence enables an increase of NGC conductivity. Addition of nanoparticles in each case resulted in a small decrease of cell viability. Nevertheless, they still maintain non-cytotoxicity and agree with the data of other researchers regarding biomaterials testing [1,2,3,40,41,42,43].

**Figure 19:**
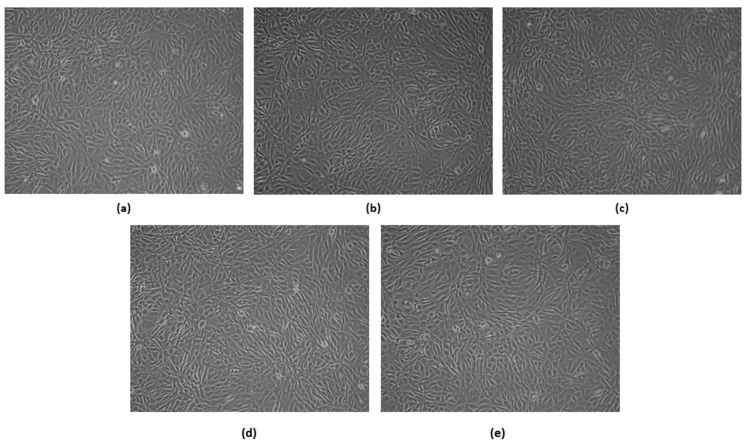
Qualitative cytotoxicity assessment of the developed samples on the 1321N1 cell line after 168 h of cell culture (40× magnification): (**a**) control; (**b**) PVP/Col/Cs; (**c**) PVP/Col/Cs/nano Au; (**d**) PVP/Col/Cs/nano Ag; (**e**) PVP/Col/Cs/nano Pt.

Astrocytes are glial cells which have many neuroprotective tasks such as stabilization, as well as regulation of the blood-brain barrier or clearing excess neurotransmitters. They also play metabolic, structural and homeostatic roles, and are responsible for promoting synapse formation. Therefore, the 132N1 cell line was used to evaluate cell-biomaterial interactions. Qualitative direct-contact cytotoxicity was carried out on the samples with four different inner layer compositions (polymers alone, and with Au, Ag, and Pt NPs). The 132N1 astrocytes cytocompatibility is shown in Figure 19. Photographs taken after 168 h of cell culture clearly demonstrate that the cellular response to biomaterials presence is positive and no morphological changes can be spotted. In all cases, cells were adhered to the substrate and proliferated forming homogenous layer of astrocytes. The cells are of normal, star-shape morphology without any abnormalities and are connected through newly formed extracellular matrix. Thus, without a doubt, a negative impact on 132N1 cells can be excluded. Moreover, the presence of the conductive biomaterial can be acknowledged as beneficial since it provides the opportunity for direct electrostimulation due to their highly conductive nature. No significant difference has been noticed between the sample, in contrast to the L929 fibroblasts, which may imply that the newly developed NGC chemical composition and architecture are more suitable for nerve tissue, and may favor peripherical nerve injury recovery by promoting glial type cell proliferation. Taking all this into account, the newly prepared biomaterials seem to meet the requirements for NGCs of the latest generation [1,2,3,40,41,42,43], and exhibit superior properties in terms of conductivity and the possibility of electrostimulation.

## 4. Conclusions

In this article, a novel approach to the issue of nerve guide conduits is proposed. A combination of two ecofriendly techniques, namely electrospinning and electrospraying enabled the preparation of hybrid, bilayer composite biomaterials, further modified with metallic nanoparticles. Semi- and final products were analyzed for their chemical structure using FT-IR and XRD techniques. Their morphology studies carried out by TEM confirmed that zero-dimensional nanomaterials of nano size were prepared. SEM photographs revealed the production of highly porous PVP nanofibers of random orientation and smooth inner composite layers with three types of NPs uniformly embedded. Thus, it was possible to select the most promising samples in terms of their composition and architecture for future applications. To evaluate their potential in peripheral nerve injury treatment and the possibility of electrostimulation to accelerate the regeneration process, NGCs were investigated for their conductivity (up to 20 m/S for PVP/Col/Cs/nano Ag), which showed that biomaterials are capable of electrical pulse transfer. Also, their long-term stability and susceptibility to enzymatic degradation has been verified to confirm their potential for continuous, several month-long treatment periods, which is sufficient for tissue recovery. Their lack of cytotoxicity has been assessed in a quantitative manner by XTT assay on L929 mouse fibroblasts, since cell viability did not fall below 70% for any case. Finally, newly developed biomaterial applicability in neurotmesis treatment was studied by analyzing cell-biomaterial interactions during direct contact with a 132N1 human glial cell culture and no morphological abnormalities were spotted. Moreover, the cells attached, spread and formed a uniform, interconnected layer. Our preliminary study has shown that the proposed, hybrid bilayer NGCs may constitute an interesting alternative to the current gold standard, namely autografts. A future study will include an in vitro electrostimulation study on Schwann cells and mesenchymal stem cells. The most promising samples will be included in an in vivo study on a small animal model (Wistar rat).

## Figures and Tables

**Table 1:** Investigated parameters of NPs synthesis.

Parameter	Range
Temperature	60–90 °C
Reaction time	20–30 min
MW power	10–25%

## Data Availability

The raw data supporting the conclusions of this article will be made available by the authors on request.
